# Is it necessary to perform measurement‐based patient‐specific quality assurance for online adaptive radiotherapy with Elekta Unity MR‐Linac?

**DOI:** 10.1002/acm2.14175

**Published:** 2023-10-10

**Authors:** Yuan Xu, Wenlong Xia, Wenting Ren, Min Ma, Kuo Men, Jianrong Dai

**Affiliations:** ^1^ Department of Radiation Oncology, National Cancer Center/National Clinical Research Center for Cancer/Cancer Hospital Chinese Academy of Medical Sciences and Peking Union Medical College Beijing China

**Keywords:** adaptive radiotherapy, Elekta Unity, MR‐linac, patient‐specific QA

## Abstract

This study aimed to investigate the necessity of measurement‐based patient‐specific quality assurance (PSQA) for online adaptive radiotherapy by analyzing measurement‐based PSQA results and calculation‐based 3D independent dose verification results with Elekta Unity MR‐Linac.

There are two workflows for Elekta Unity enabled in the treatment planning system: adapt to position (ATP) and adapt to shape (ATS). ATP plans are those which have relatively slighter shifts from reference plans by adjusting beam shapes or weights, whereas ATS plans are the new plans optimized from the beginning with probable re‐contouring targets and organs‐at‐risk. PSQA gamma passing rates were measured using an MR‐compatible ArcCHECK diode array for 78 reference plans and corresponding 208 adaptive plans (129 ATP plans and 79 ATS plans) of Elekta Unity. Subsequently, the relationships between ATP, or ATS plans and reference plans were evaluated separately. The Pearson's r correlation coefficients between ATP or ATS adaptive plans and corresponding reference plans were also characterized using regression analysis. Moreover, the Bland–Altman plot method was used to describe the agreement of PSQA results between ATP or ATS adaptive plans and reference plans. Additionally, Monte Carlo‐based independent dose verification software ArcherQA was used to perform secondary dose check for adaptive plans.

For ArcCHECK measurements, the average gamma passing rates (ArcCHECK vs. TPS) of PSQA (3%/2 mm criterion) were 99.51% ± 0.88% and 99.43% ± 0.54% for ATP and ATS plans, respectively, which were higher than the corresponding reference plans 99.34% ± 1.04% (*p* < 0.05) and 99.20% ± 0.71% (*p* < 0.05), respectively. The Pearson's r correlation coefficients were 0.720 between ATP and reference plans and 0.300 between ATS and reference plans with ArcCHECK, respectively. Furthermore, >95% of data points of differences between both ATP and ATS plans and reference plans were within ±2σ (standard deviation) of the mean difference between adaptive and reference plans with ArcCHECK measurements. With ArcherQA calculation, the average gamma passing rates (ArcherQA vs. TPS) were 98.23% ± 1.64% and 98.15% ± 1.07% for ATP and ATS adaptive plans, separately.

It might be unnecessary to perform measurement‐based PSQA for both ATP and ATS adaptive plans for Unity if the gamma passing rates of both measurements of corresponding reference plans and independent dose verification of adaptive plans have high gamma passing rates. Periodic machine QA and verification of adaptive plans were recommended to ensure treatment safety.

## 1 INTRODUCTION

Radiation therapy requires precise treatment to effectively treat oncology patients. Image guidance is commonly used to monitor inter‐fraction and intra‐fraction motions of targets or organs–at‐risk (OARs) during radiotherapy.^1^, ^2^ MR‐guided radiotherapy (MRgRT) has recently been introduced and has become a promising option for image‐guided radiation therapy.^3^, ^4^ MRgRT has numerous advantages over traditional x‐ray cone‐beam computed tomography, such as high soft‐tissue contrast, functional imaging, real‐time imaging, and no additional radiation exposure.^5^, ^6^ This enables adapting to anatomical variations and treatment plans based on daily MR imaging, eventually leading to tumor gating or tracking during treatment.^7^, ^8^

There are two types of MR‐guided treatment equipment: radioactive isotope‐based^9^ or linac‐based.^10^ Elekta Unity MR‐Linac (Elekta, Crawley, UK) integrated a 7 MV linear accelerator with a 1.5 T MR scanner for adaptive radiotherapy with daily MR imaging.^11^, ^12^ The Monaco treatment planning system (TPS) (version 5.4, Elekta, Crawley, UK) provides two adaptive workflows for Unity: adapt to position (ATP) and adapt to shape (ATS).^13^, ^14^ In both workflows, a reference plan is initially optimized with pre‐treatment planning CT. In ATP, the beam weights or shapes of the reference plan are optimized based on the rigid registration of pre‐treatment CT with online MR.^14^ Contouring of targets or OARs cannot be edited in the ATP workflow. In ATS, however, contouring can be manually edited based on deformable registration results with daily MR if necessary and a new treatment plan is then created and optimized from the beginning with newly contoured targets and OARs.^14^

Patient‐specific quality assurance (PSQA) is a standard step in the radiotherapy workflow. It helps to identify any discrepancies between the radiation dose that is calculated by the treatment planning system (TPS) and the dose that is delivered by the radiotherapy system.^15^, ^16^ This step is important to ensure the safety and accuracy of radiation therapy. PSQA is particularly important for intensity‐modulated radiotherapy (IMRT) plans due to their high complexity. In fact, the American Association of Physicists in Medicine (AAPM) Task Group 218^17^ recommends that PSQA be performed for all IMRT plans. One commonly used PSQA method is phantom measurement, which involves placing a homogeneous phantom on the treatment couch and delivering the planned dose to the phantom.^18^, ^19^ Detectors located inside the phantom measure the radiation dose, which is then compared to the dose calculated by the TPS based on the phantom image. Elekta Unity also utilizes phantom measurement to verify the plan quality of both reference and adaptive plans.^20^ However, for Elekta Unity's ATP and ATS adaptive plans, the planning, and optimization are performed with patients lying on the treatment couch. It is not convenient and efficient for treatment team to verify adaptive plans with phantom measurement before treatment because patients are receiving online treatment. In clinical practice, phantom measurement of online adaptive plans is usually performed after treatment, which cannot effectively detect any possible problems before treatment.

As mentioned earlier, a reference plan is created offline for Elekta Unity and can be verified with phantom measurement before patient treatment. Both ATP and ATS adaptive plans are optimized based on reference plans, which may have an impact on the PSQA results. Strand et al. analyzed gamma passing rates of 68 reference plans and 512 adaptive plans of Unity using the statistical process control (SPC) method.^21^ They reported that the one‐sided process capability ratio was 1.403 and 0.940 for the gamma passing rates of ATP and ATS plans compared to reference plans, respectively. This indicates that the PSQA results of ATP plans were within the variability of reference plans while ATS plans were not.^21^ However, the relationships between adaptive plans and reference plans were not very clear and require further study.

Calculation‐based independent dose verification is a possible solution for online adaptive radiotherapy. AAPM TG219 reports recommended performing independent dose check for all IMRT or volumetric‐modulated arc therapy (VMAT) plans.^22^ For MR‐linac, the dose calculation tends to become complicated with the existence of magnetic field. Therefore, only a few dose/MU check programs for Unity MR‐linac were commercially available so far.^23^, ^24^ ArcherQA (Wisdom Technology Company Limited, Hefei, China) is a GPU‐accelerated Monte Carlo‐based independent dose verification software.^25^ Recently, a machine model of Unity MR‐linac was built and commissioned with ArcherQA, which carefully considered the impact of 1.5 T magnetic field on dose distribution.^26^ Adaptive plans were recalculated with ArcherQA, and compared with measurements using 3D gamma analysis in this study. Additionally, we explored the relationships between PSQA results (ArcCHECK vs. TPS) of adaptive plans and their corresponding reference plans, combined with independent dose verification results (ArcherQA vs. TPS), to determine whether it is necessary to perform PSQA for Unity online adaptive radiotherapy using statistical methods. Due to the different characteristics of ATP and ATS plans, the relationships between the PSQA results of these plans and their corresponding reference plans were analyzed separately. More details will be presented in this manuscript.

## 2 MATERIALS AND METHODS

### 2.1 Patient and plan information

This study received approval from the ethical review board of our institution and informed consent was waived. In this retrospective study, we analyzed 78 reference plans and 208 adaptive plans (129 ATP plans and 79 ATS plans) delivered using the Elekta Unity MR‐Linac (Elekta, Crawley, UK) over the past year. To avoid the impact of a single patient with many fractions, up to three fractions’ adaptive plans (initial, medium, and final fractions) were enrolled for each patient. For ATP plans, there were 37 head and neck cases, 9 thorax cases, 56 abdomen cases, and 27 pelvic cases. Conversely, all ATS plans were abdominal and pelvic cases, mostly for prostate cancer. All patients were treated with a 7 MV MR‐Linac Elekta Unity in the flattening filter‐free mode, and IMRT plans were optimized using the Monaco TPS (version 5.4, Elekta, Crawley, UK).

### 2.2 Phantom measurements

**FIGURE 1:**
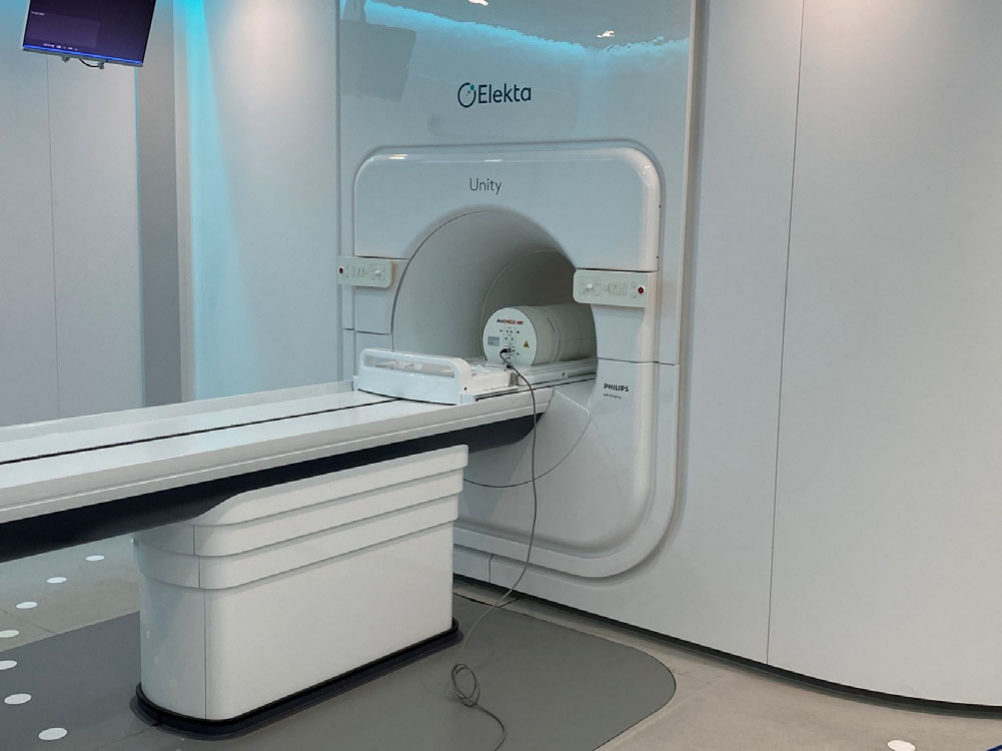
Installation of a MR‐compatible ArcCHECK phantom on the treatment couch of Unity.

Each plan was recalculated using the CT image of an MR‐compatible ArcCHECK phantom (Sun Nuclear Corporation, Melbourne, USA) with Monte Carlo algorithm (GPUMCD) and dose grid of 0.2 mm × 0.2 mm × 0.2 mm. The ArcCHECK phantom was then mounted on the treatment couch using a holder to immobilize it, as shown in Figure 1. The data was collected and analyzed using SNC patient software (version 8.2, Sun Nuclear Corporation, Melbourne, USA). The gamma passing rate (ArcCHECK vs. TPS) was used to identify discrepancies between TPS calculations and phantom measurements. As recommended by AAPM report TG218,^17^ a criterion of 2 mm/3% with global normalization and a threshold of 10% was implemented.

### 2.3 Independent dose verification with ArcherQA

All adaptive plans were transmitted to independent dose verification software ArcherQA (Wisdom Technology Company Limited, Hefei, China) to re‐calculate the dose distribution. Monte Carlo algorithm was used for simulating motions of electrons and photons and their interaction with other particles. Magnetic field was considered using a special transport model, and model of Unity was built using commissioning data from our machine and was validated with clinical cases.^26^ 3D gamma passing rate (ArcherQA vs. TPS) were compared between dose calculated with Monaco TPS and ArcherQA with a criterion of 2 mm/3% with global normalization and a threshold of 10%.

### 2.4 Statistical significance

Statistical analyses of gamma passing rates between adaptive plans and corresponding reference plans were performed using SPSS (version 20.0, IBM, New York, USA). First, normality of data was tested with Kolmogorov–Smirnov test. Subsequently, paired samples *t*‐test was used for data with a normal distribution; otherwise, nonparametric Wilcoxon signed‐rank test was utilized to test significance for parameters. A *p*‐value of <0.05 was considered statistically significant.

### 2.5 Correlation coefficient

The correlation between gamma passing rates (ArcCHECK vs. TPS) of ATP or ATS plans and those of reference plans was analyzed using the regression method. The adjusted coefficient of determination *R* ^2^ and Pearson's r correlation coefficient were used to characterize the correlation. The value of Pearson's r ranges from −1 to 1; an absolute value of r larger than 0.7 indicates a strong correlation, 0.4−0.7 indicates a moderate correlation, and <0.4 indicates a weak correlation.^27^

### 2.6 Bland–Altman analysis

While the correlation coefficient can only indicate the relationship between variables, it cannot provide information on their differences. Therefore, the correlation coefficient cannot be used to evaluate the comparability of two variables. To assess the agreement between ATP or ATS plans and reference plans, the widely used Bland–Altman plot method was employed.^28^, ^29^, ^30^ The method is used in the medical field to evaluate the agreement between two different quantitative measurement methods. In this study, the Bland–Altman plot method was utilized to characterize the agreement between ATP or ATS plans and reference plans. The differences between the gamma passing rates (ArcCHECK vs. TPS) of ATP or ATS adaptive plans and the reference plans were plotted as scatters, while the average difference was plotted as a horizontal line. The upper and lower limit lines were drawn as the average difference ±2σ (standard deviation). It was recommended that 95% of data points should lie within the range of ±2σ around the mean differences.^30^

## 3 RESULTS

### 3.1 Statistical significance

**FIGURE 2:**
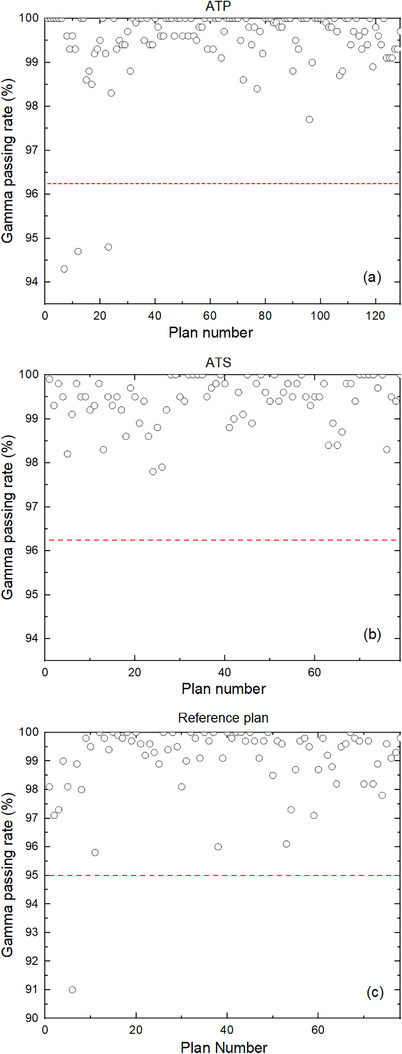
Gamma passing rates of measurement‐based‐PSQA: ATP (a), ATS (b), and reference plans (c). ATP, adapt to position; ATS, adapt to shape; PSQA, patient‐specific quality assurance.

The gamma passing rates (ArcCHECK vs. TPS) for MR‐compatible ArcCHECK 129 ATP plans, 79 ATS plans, and 78 reference plans are presented in Figure 2. The average gamma passing rates (ArcCHECK vs. TPS) for ATP and ATS adaptive plans are 99.51% ± 0.88% and 99.43% ± 0.54%, respectively, which are significantly higher than the corresponding reference plans of 99.34% ± 1.04% (*p* < 0.05) and 99.20% ± 0.71% (*p* < 0.05). The gamma passing rates (ArcCHECK vs. TPS) of all ATS plans were higher than 95% (tolerance limit recommended by AAPM reports TG218), whereas gamma passing rates (ArcCHECK vs. TPS) of only 3 ATP plans were lower than 95%.

### 3.2 Correlation coefficient

**FIGURE 3:**
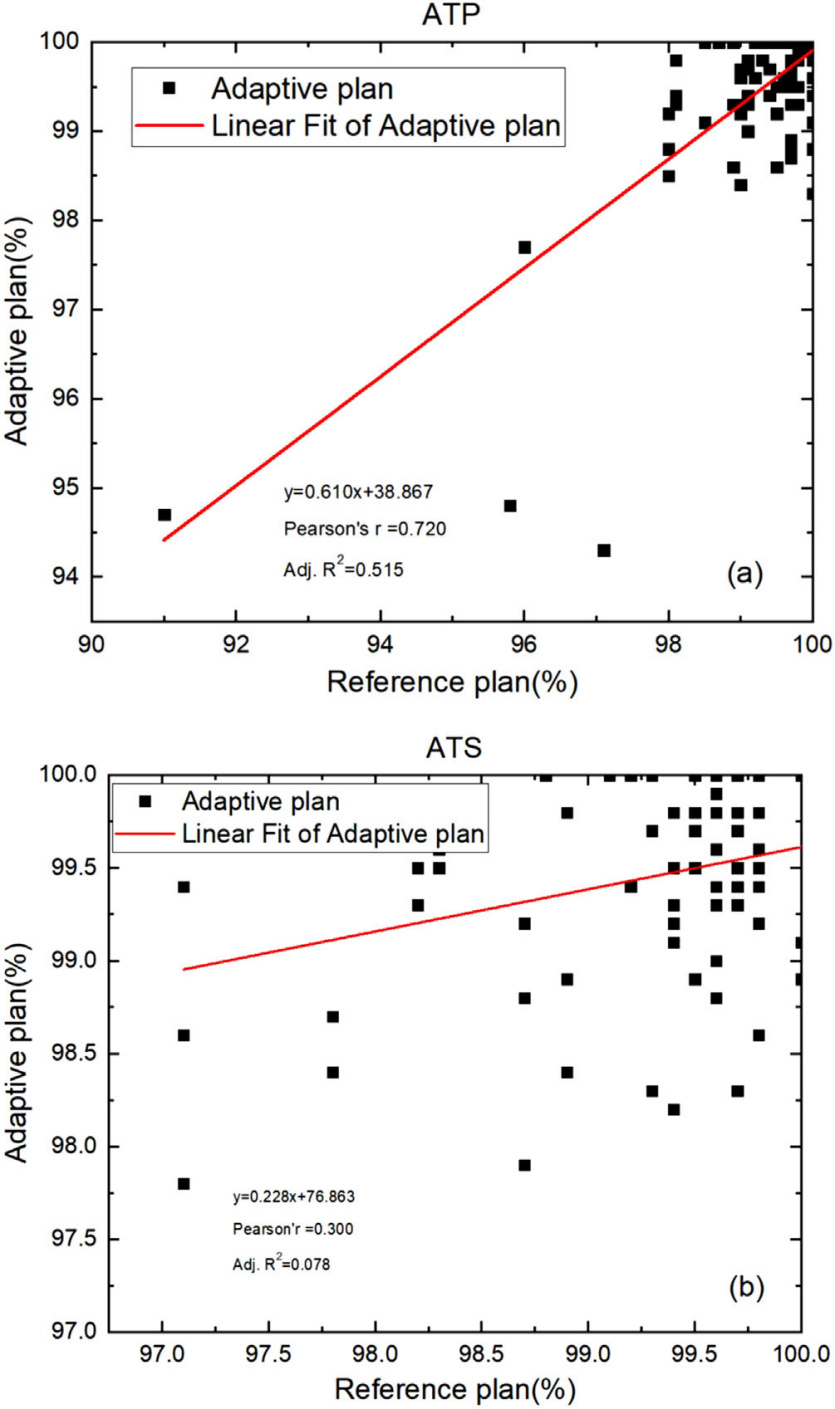
Correlation analysis of gamma passing rates between ATP plans (a) or ATS plans (b) and reference plans. ATP, adapt to position; ATS, adapt to shape.

Correlation analysis of gamma passing rates (ArcCHECK vs. TPS) between ATP or ATS plans and reference plans was performed using the regression method. Figure 3 shows that the Pearson's r correlation coefficients are 0.720 and 0.300 for ATP and ATS plans, respectively. The adjusted *R* ^2^ values are 0.515 and 0.078 for ATP and ATS plans, respectively. These results indicate a strong correlation between ATP plans and the corresponding reference plans but a weak correlation between ATS plans and the corresponding reference plans.

### 3.3 Bland–Altman plot

**FIGURE 4:**
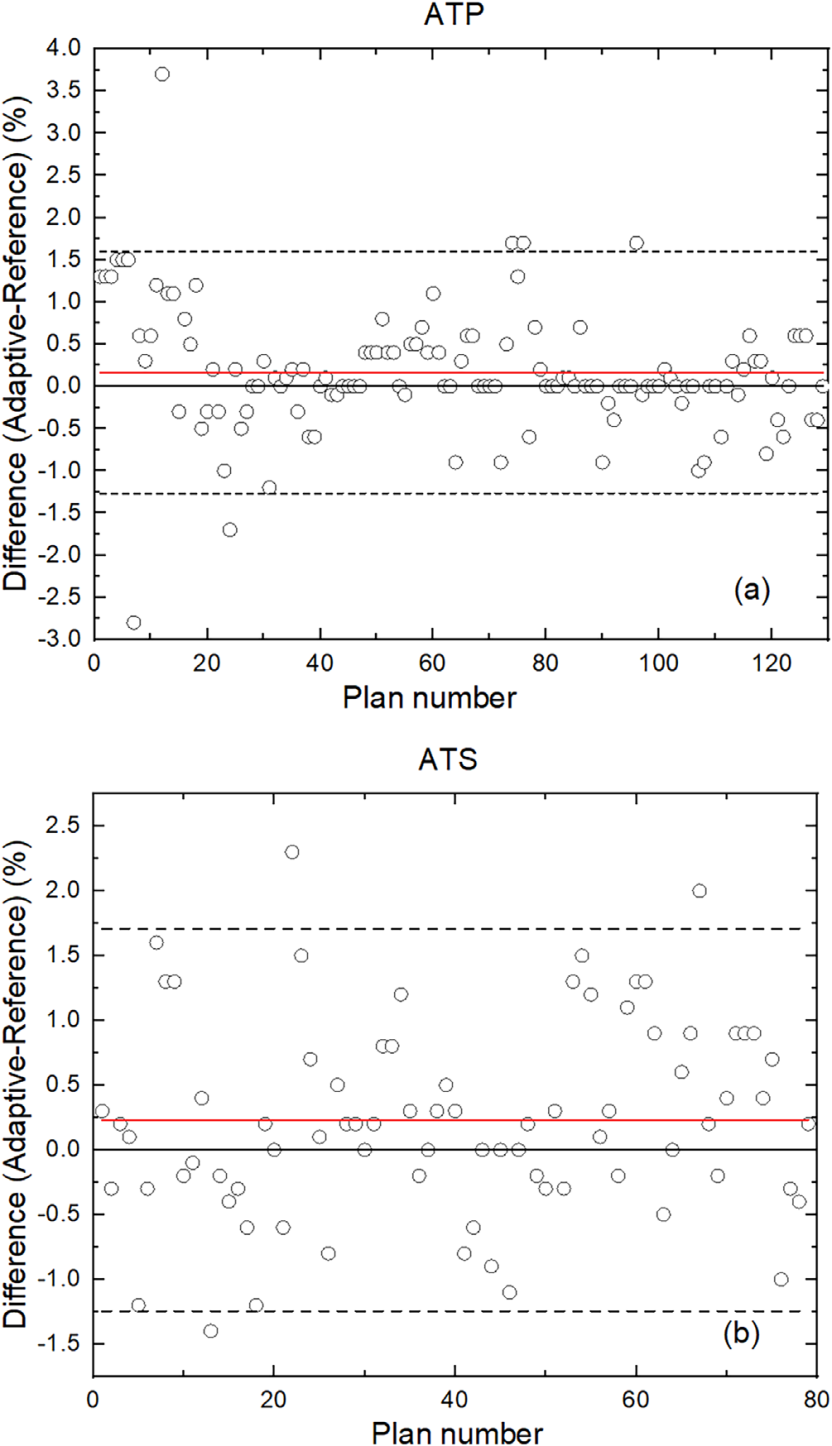
Bland–Altman plot of ATP plans (a) and ATS plans (b). (Scatters: difference between adaptive and reference plans; red solid line: mean difference; black solid line: zero; black dash line: mean ±2σ). ATP, adapt to position; ATS, adapt to shape.

Figure 4 shows the Bland–Altman plots of the differences between the gamma passing rates (ArcCHECK vs. TPS) of adaptive plans and reference plans for ATP and ATS plans. The mean difference and mean difference ±2σ are drawn as three horizontal lines. For ATP plans, the maximum difference was 3.7%, and 123 out of 129 (95.35%) data points fell within the range of ±2σ around the mean difference. For ATS plans, the maximum difference was 2.3%, and 76 out of 79 (96.20%) data points were within the variation of ± 2σ. Therefore, both ATP, and ATS plans exhibited good agreement with their corresponding reference plans, as more than 95% of the data points fell within the ±2σ lines.

### 3.4 Independent dose verification with ArcherQA

**FIGURE 5:**
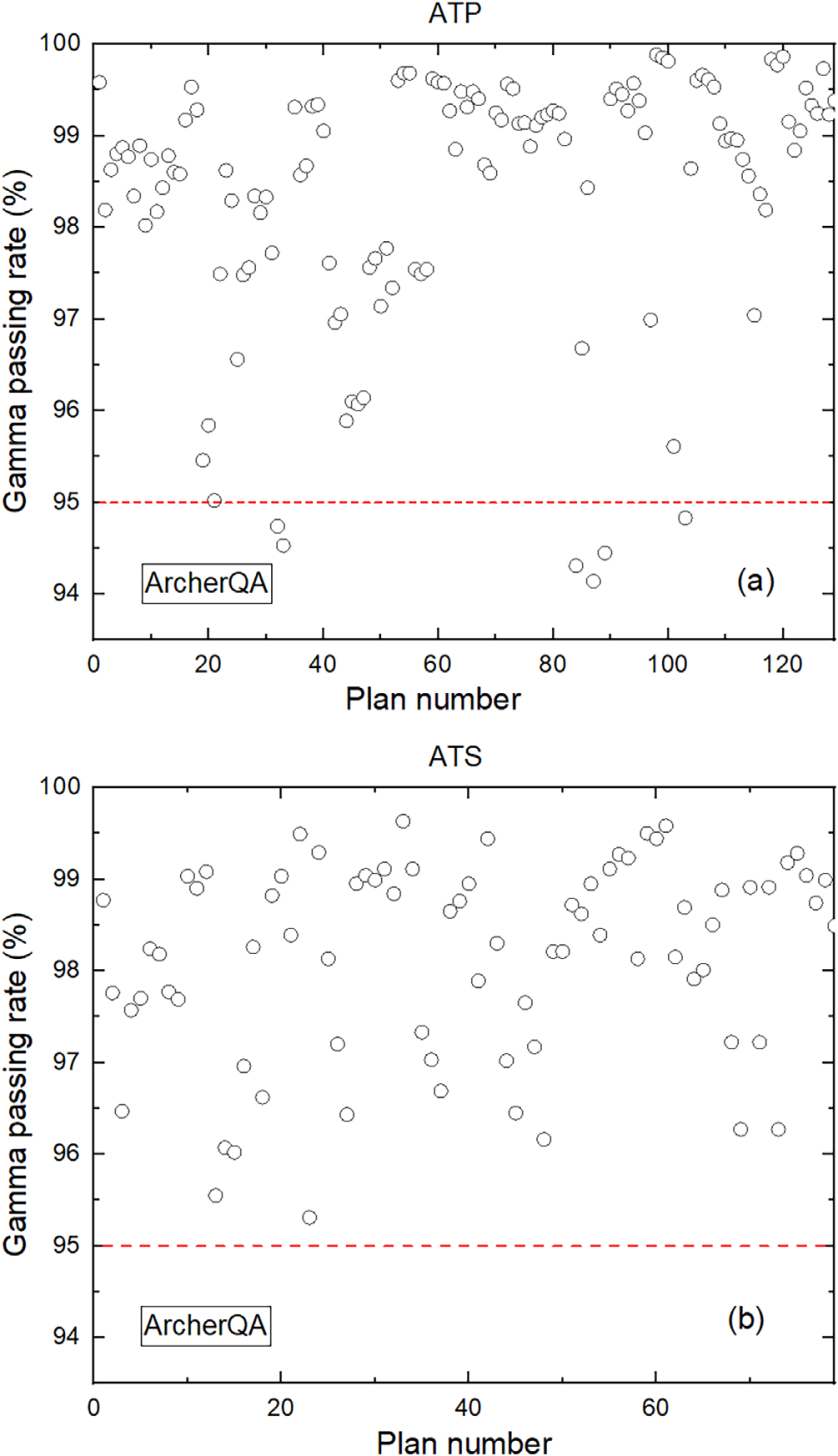
Gamma passing rates of independent dose verification: ATP (a) and ATS (b). ATP, adapt to position; ATS, adapt to shape.

The 3D gamma passing rates (ArcherQA vs. TPS) between doses calculated with TPS and ArcherQA of ATP and ATS adaptive plans are illustrated in Figure 5 a and b, separately. The mean gamma passing rates (ArcherQA vs. TPS) (2 mm/3% criterion) were 98.23% ± 1.64% and 98.15% ± 1.07% for ATP and ATS adaptive plans, respectively. Gamma passing rates (ArcherQA vs. TPS) of 92.2% ATP plans and 100% ATS plans were higher than 95%.

## 4 DISCUSSION

MRgRT has great potential for online adaptive radiotherapy, allowing for real‐time adjustments to account for anatomical changes during treatment. However, traditional phantom measurement‐based PSQA methods cannot be used to verify the accuracy of adaptive plans, as these plans are optimized while patients are on the treatment couch. One possible solution is to validate adaptive plans using software calculation‐based PSQA methods with different algorithms.^31^, ^32^ This can be realized with ArcherQA independent dose verification module, which considers the influence of magnetic field.^26^ For Unity MR‐Linac, the reference plan is optimized as a foundation for ATP or ATS plans and can be verified with a phantom before patient treatment. Understanding the relationship between PSQA results of ATP or ATS plans and reference plans combined with the results of independent dose verification is necessary to explore the necessity of measurement‐based PSQA for adaptive plans. Zhao et al. discussed the necessity of measurement‐based PSQA for online adaptive radiotherapy with conventional accelerator Varian Ethos, and Mobius3D was used to perform 3D dose verification.^33^ They concluded that measurement‐based PSQA might not be required for every adaptive plan. In this study, the mean gamma passing rates (ArcCHECK vs. TPS) for both ATP and ATS adaptive plans were high, exceeding 99%, and significantly higher than corresponding reference plans (*p* < 0.05). Gamma passing rates (ArcCHECK vs. TPS) of only three adaptive plans were lower than 95%, and high gamma passing rates (ArcCHECK vs. TPS) for Unity plans might be because IMRT technique was used for treatment planning, which normally had a relatively higher gamma passing rate compared with VMAT, and machine QA were performed appropriately. The Pearson's r correlation coefficients showed a strong correlation between the PSQA results of ATP plans and reference plans, and a weak correlation between those of ATS plans and reference plans. This is consistent with the fact that ATP adaptive plans are slight adjustments of beam weights and shapes of reference plans, whereas ATS plans are new plans created only by utilizing the original optimization set‐up of reference plans. Similar results were obtained with SPC methods, which showed that ATP plans were within the variability of reference plans, whereas ATS plans were not.^21^

The Bland–Altman plots showed that both ATP and ATS plans were in good agreement with their corresponding reference plans. The maximum difference between the gamma passing rate (ArcCHECK vs. TPS) of the adaptive plans and their reference plans was 3.7%. The patient in this treatment plan had partial breast cancer and received tangential field radiotherapy. However, the target was relatively far away from the center of the phantom. Due to the fixed holder of the MR‐compatible ArcCHECK phantom and the inability to move the treatment couch in lateral directions, only the low dose region of partial breast cancer radiation could be measured with detectors in the ArcCHECK phantom, which may have led to a larger error in the measurement. For ArcherQA, gamma passing rate (ArcherQA vs. TPS) lower than 95% were mainly represented rectum cancer. This was because the field size was relatively large (about 20 cm) for rectum cancer. There could be some discrepancies for the beam modeling between TPS and ArcherQA in wide field size.

The lowest gamma passing rate (ArcCHECK vs. TPS) was 94.3% and 97.8% for ATP and ATS adaptive plans, separately, when gamma passing rates of both measurements of reference plans and independent dose verification of adaptive plans exceeded 95% (2 mm/3%). With this criterion, 91.5% ATP plans and 100% ATS plans could pass it. Overall, considering gamma passing rates of both measurements of reference plans and independent dose verification of adaptive plans, if both of them have a high gamma passing rates (> 95%), provides us confidence that adaptive plans are acceptable for treatment. For plans with gamma passing rate of reference plan or ArcherQA calculation lower than 95%, it is recommended to perform post treatment QA measurements or re‐optimize adaptive plans to ensure treatment safety. It should be noted that mainly prostate cancer was included for analysis for ATS adaptive plans because the ATS workflow was mostly implemented for prostate cancer in our institute, which may have resulted in biased results for the ATS adaptive plans. Furthermore, a periodic machine QA and sampling validation of adaptive plans are recommended to ensure treatment safety.

## 5 CONCLUSIONS

The study investigated the correlation between gamma passing rates (ArcCHECK vs. TPS) of Elekta Unity MR‐Linac's ATP and ATS adaptive plans and corresponding reference plans and performed an additional independent dose verification for adaptive plans. The results showed that the gamma passing rates (ArcCHECK vs. TPS) of ATP plans had a strong correlation with reference plans, while the correlation between ATS plans and reference plans was weak. Additionally, both ATP and ATS adaptive plans were in good agreement with the reference plans. Therefore, the study concludes that there might be no need to perform measurement‐based PSQA for Unity's adaptive plans if the corresponding reference plans and independent dose verification have high gamma passing rates exceeding 95%. However, it is recommended to perform machine QA and sampling validation of adaptive plans to ensure treatment safety.

## AUTHOR CONTRIBUTIONS

Yuan Xu: conception design, collected the data, manuscript writing. Wenlong Xia and Wenting Ren: dosimetric verification with phantom measurements. Min Ma: statistical analysis. Jianrong Dai and Kuo Men: supervised the study.

## CONFLICT OF INTEREST STATEMENT

There is no conflict of interest to declare.
